# Elimination of fibrin γ-chain cross-linking by FXIIIa increases pulmonary embolism arising from murine inferior vena cava thrombi

**DOI:** 10.1073/pnas.2103226118

**Published:** 2021-06-28

**Authors:** Cédric Duval, Adomas Baranauskas, Tímea Feller, Majid Ali, Lih T. Cheah, Nadira Y. Yuldasheva, Stephen R. Baker, Helen R. McPherson, Zaher Raslan, Marc A. Bailey, Richard M. Cubbon, Simon D. Connell, Ramzi A. Ajjan, Helen Philippou, Khalid M. Naseem, Victoria C. Ridger, Robert A. S. Ariëns

**Affiliations:** ^a^Leeds Thrombosis Collective, Discovery & Translational Science Department, Leeds Institute of Cardiovascular and Metabolic Medicine, University of Leeds, Leeds LS2 9NL, United Kingdom;; ^b^School of Physics and Astronomy, University of Leeds, Leeds LS2 3AR, United Kingdom;; ^c^Department of Infection, Immunity and Cardiovascular Disease, The Medical School, University of Sheffield, Sheffield S10 2RX, United Kingdom

**Keywords:** fibrin, Factor XIII, thromboembolism, mechanical properties, clot structure

## Abstract

The onset of venous thromboembolism, including pulmonary embolism, represents a significant health burden affecting more than 1 million people annually worldwide. Current treatment options are based on anticoagulation, which is suboptimal for preventing further embolic events. In order to develop better treatments for thromboembolism, we sought to understand the structural and mechanical properties of blood clots and how this influences embolism in vivo. We developed a murine model in which fibrin γ-chain cross-linking by activated Factor XIII is eliminated (FGG3X) and applied methods to study thromboembolism at whole-body and organ levels. We show that FGG3X mice have a normal phenotype, with overall coagulation parameters and platelet aggregation and function largely unaffected, except for total inhibition of fibrin γ-chain cross-linking. Elimination of fibrin γ-chain cross-linking resulted in thrombi with reduced strength that were prone to fragmentation. Analysis of embolism in vivo using Xtreme optical imaging and light sheet microscopy demonstrated that the elimination of fibrin γ-chain cross-linking resulted in increased embolization without affecting clot size or lysis. Our findings point to a central previously unrecognized role for fibrin γ-chain cross-linking in clot stability. They also indirectly indicate mechanistic targets for the prevention of thrombosis through selective modulation of fibrin α-chain but not γ-chain cross-linking by activated Factor XIII to reduce thrombus size and burden, while maintaining clot stability and preventing embolism.

## Significance

Pulmonary embolism and stroke are thromboembolic diseases affecting >1 million people annually worldwide. Thromboembolism involves clot fragments affecting vital downstream organs such as the lung or brain. The mechanisms underpinning thromboembolism are not understood and require clarification in order to devise more effective treatments. Here, we developed a thromboembolism protocol supported by state-of-the-art in vivo imaging, coupled to a genetically modified murine model of reduced clot strength caused by mutations in the cross-linking sites of fibrin, which provides the mechanical scaffold of the clot. We find that fibrin γ-chain cross-linking is essential for clot stability and reduces embolism. These findings introduce an important concept indicating that maintenance of clot stability during thrombosis treatment is essential to prevent thromboembolism.

Thrombosis is complicated by life-threatening embolic events, caused by parts of an intravascular blood clot breaking off and traveling downstream to block other blood vessels supplying critical organs. Thromboembolism occurs in both the venous and arterial circulation and is associated with life-threatening pulmonary embolism (PE) (1) and ischemic stroke (2). PE occurs when thrombi in the deep veins of the limb embolise and passage with the flowing blood through the inferior vena cava, the right atrium, and ventricle of the heart to the lungs (3), causing pulmonary hypertension and respiratory failure (4). Venous thromboembolism (VTE), comprising deep vein thrombosis (DVT) and PE, which globally affects over 1 million people each year (1), results in substantial healthcare costs (5) and is a major cause of death worldwide (1, 6). Thromboembolism is clinically challenging to treat. Anticoagulation with vitamin K antagonists or direct oral anticoagulants are used to treat VTE and prophylactically to prevent VTE recurrence (7). In PE, localized thrombolysis with plasminogen activators is challenging and often only used as a last resort to help remove emboli resistant to anticoagulation (8). Improvements in treatment and prevention of thromboembolic disorders are therefore urgently needed.

Recent studies indicate that structural and functional properties of the clot could be critical in thromboembolism, and these parameters may offer novel areas for therapeutic intervention. Hypofibrinolysis was reported to increase the risk of a first DVT (9), while changes in clot properties (increased clot formation rate and fiber density, reduced fibrinolysis) promoted the recurrence of DVT (10). Abnormalities in the establishment of clot viscoelastic properties have been shown to increase risk of PE (11), and reduced clot elastic modulus has been associated with VTE recurrence (12). However, the exact mechanisms linking altered clot properties to increased thromboembolic risk are unclear, and therefore, treatment options remain limited and rely on dissolution of fibrin networks and prevention of future clot formation, which carry significant risk of bleeding events.

A key regulator of clot mechanical properties is coagulation Factor XIII (FXIII), a protransglutaminase that is converted into the active transglutaminase (FXIIIa) by thrombin (13). FXIIIa catalyzes the formation of γ-glutamyl-ε-lysine isopeptide bonds between adjacent molecules within the fibrin fibers to substantially increase elastic moduli and reduce storage moduli of both individual fibrin fibers and fibrin networks (14–17), therefore making the clot more elastic and less viscous. FXIIIa cross-links fibrin γ-chain residues Q398 and Q399 with K406 (18, 19), and α-chain residues Q221, Q237, Q328, and Q366 with numerous lysine residues (20–22). We previously demonstrated a critical role for γ-chain cross-linking by FXIIIa in generating clot viscoelastic properties, in particular by increasing the elastic or Young’s modulus, using a human recombinant fibrinogen γ-3X (γ-Q398N/Q399N/K406R) mutant of the essential γ-chain cross-linking sites (23–25).

The fibrin γ-chain cross-linking sites for FXIIIa are highly conserved, and based on our previous in vitro data (23–25), we have now generated a genetically modified mouse in which the fibrin γ-chain cross-linking sites are mutated (FGG3X) to understand the role of fibrin fiber cross-linking in predisposition to embolic disease. We confirm the importance of γ-chain cross-linking in enhancing clot mechanical properties in vivo. Furthermore, using two protocols to study VTE, we demonstrate that lack of γ-chain cross-linking by FXIIIa increases thromboembolism using advanced whole-body and whole-organ imaging. We further show that fibrin fibers lacking γ-chain cross-linking are more prone to rupture at lower stress. These data indicate that fibrin γ-chain cross-linking enhances the resistance of fibrin fibers to rupture, consequently reducing clot fragmentation and thromboembolism.

## Results

### Generation and Characterization of the FGG3X Model.

**Fig. 1.**
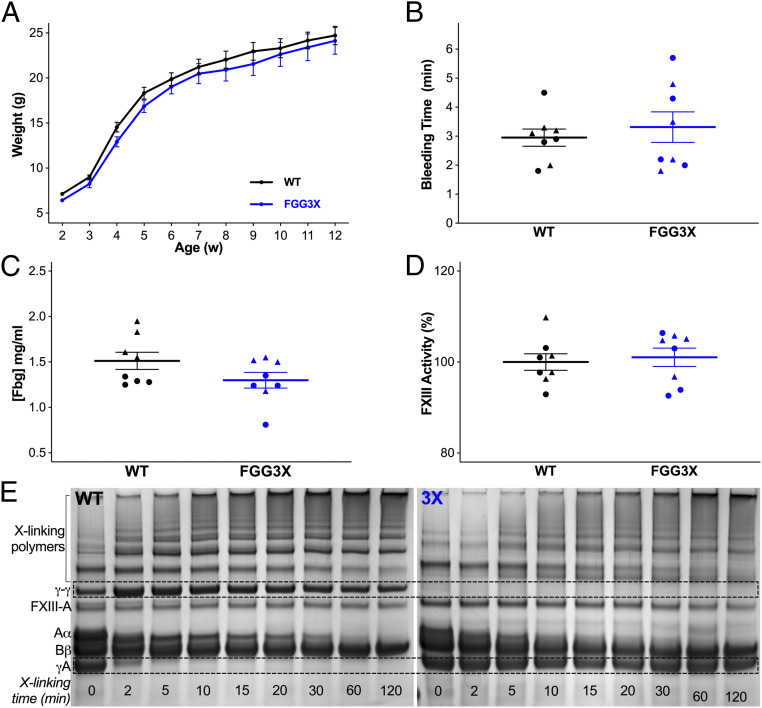
Mouse model characterization. WT and FGG3X mice showed no difference in growth, measured by weight (*A*), over the first 12 wk (*n* = 10). Tail sectioning bleeding time (*B*), plasma fibrinogen concentration (*C*), and plasma FXIII activity measured by thrombin- and CaCl_2_-induced (2 U/mL and 10 mM) pentylamine-biotin incorporation to fibrin (*D*) were not different between these two mouse strains. Fibrinogen, purified from plasma by ammonium sulfate precipitation, was assayed for cross-linking of 5 μg fibrin by FXIII (10 μg/mL) activated with thrombin and CaCl_2_ (0.5 U/mL and 10 mM) (*E*) and showed full inhibition of fibrin γ-chain cross-linking (no production of γ-γ dimers and continued presence of γ-monomers) in FGG3X, compared to WT, while the α-chain cross-linking was not affected, as shown by the normal disappearance of α-monomers and appearance of polymers (*n* = 4). *n* = 8 (*A–D*) and 4 (*E*); [▲] males, [●] females. The data are presented as mean ± SEM and analyzed by two-way ANOVA test (*A*) and Mann–Whitney *U* test (*B–D*).

FGG3X mice were generated on a C57BL/6 background, in which the conserved fibrinogen γ-chain cross-linking residues Q398/Q399 and K406 (*SI Appendix*, Fig. S1) were mutated to N and R residues, respectively, using homologous recombination (*SI Appendix*, Fig. S2). These mutations recapitulate the mutations generated in our previously described recombinant human fibrinogen variant (γ-3X), tested in vitro, which prevents γ-chain cross-linking by FXIIIa (23). In comparison to wild type (WT), FGG3X mice showed no difference in growth over 12 wk after birth (Fig. 1*A*). In addition, no differences in bleeding time (Fig. 1*B*), fibrinogen antigen level (Fig. 1*C*), FXIII activity (Fig. 1*D*), and whole blood counts (Table 1) were observed, while there were no rebleeding events in both WT and FGG3X mice. However, FGG3X mice showed a total elimination of fibrin γ-chain cross-linking, while α-chain cross-linking remained unaffected (Fig. 1*E*), in agreement with our previous in vitro human recombinant fibrinogen studies (23).

**Table 1.** Hematological parameters in WT and FGG3X mice

Parameters	WT	FGG3X	*P* values*
RBC (10^12^/mL)	5.34 ± 0.05	5.51 ± 0.07	0.127
HGB (g/dL)	8.83 ± 0.11	9.08 ± 0.11	0.226
HCT (%)	29.91 ± 0.32	29.86 ± 0.33	0.940
PLT (10^11^/mL)	5.99 ± 0.71	7.79 ± 0.47	0.083
WBC (10^9^/mL)	3.33 ± 0.51	3.14 ± 0.22	0.775
LYM (%)	81.89 ± 1.23	84.41 ± 0.86	0.111
NEU (%)	8.48 ± 0.77	8.10 ± 0.53	0.857
MXD (%)	9.71 ± 0.71	7.58 ± 0.43	0.066

RBC: red blood cells; HGB: hemoglobin, HCT: hematocrit; PLT: platelets; WBC: white blood cells; LYM: lymphocytes; NEU: neutrophils; and MXD: mixed white blood cells.

* Data analyzed by Mann–Whitney *U* test. *n* = 8.

### Whole–Blood Clot Firmness Is Decreased in FGG3X Mice, while Clot Contraction Is Unaffected.

**Fig. 2.**
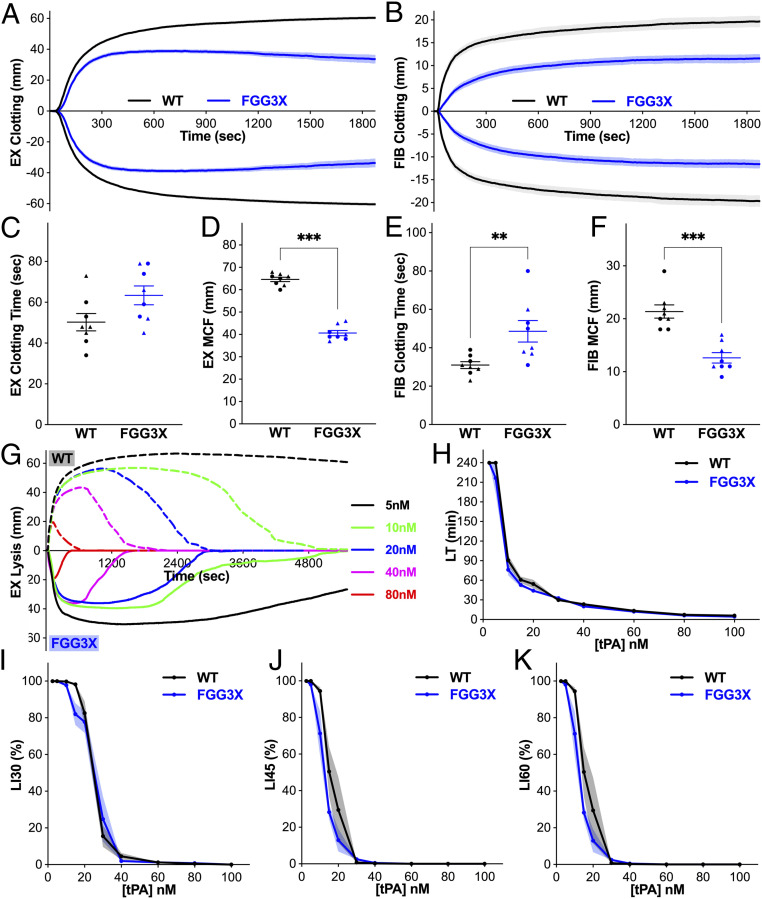
FGG3X mice form blood clots that are less firm than WT mice. EXTEM (7 μL + 3 μL TBS) (*A*, *C*, and *D*) and FIBTEM (7 μL + 3 μL TBS) (*B*, *E*, and *F*) analysis of whole blood (103 μL + 7 μL STARTEM) samples showed that clotting time (*C* and *E*) was not significantly different between WT and FGG3X mice, while maximum clot firmness (*D* and *F*) was significantly decreased in FGG3X mice compared to WT mice. EXTEM in the presence of a range of tPA concentration (2.5 to 100 nM) (*G*) showed that lysis time (*H*), % lysis at 30 (*I*), 45 (*J*), and 60 min (*K*), were similar between WT (dashed lines) and FGG3X mice (solid lines). *n* = 8; [▲] males, [●] females. The data analyzed are presented as mean ± SEM and analyzed by Mann–Whitney *U* test; **P < 0.01, ***P < 0.001.

We investigated the effects of the lack of fibrin γ-chain cross-linking on whole–blood clot formation and stability ex vivo. Rotational thromboelastometry (ROTEM) analysis was performed using both EXTEM (tissue factor Fig. 2*A*) and FIBTEM (platelet inhibitor cytochalasin D, Fig. 2*B*) protocols to investigate the role of fibrin γ-chain cross-linking in the presence or absence of platelet activity, respectively. Whole–blood clotting time was prolonged by 1.3-fold in EXTEM (Fig. 2*C*) and 1.6-fold (*P* < 0.01) in FIBTEM (Fig. 2*E*) for FGG3X compared to WT mice, indicating a role for γ-chain cross-linking in early stabilization of nascent fibrin fibers. Maximum clot firmness was decreased significantly (*P* < 0.001) by 1.6- and 1.7-fold in EXTEM (Fig. 2*D*) and FIBTEM (Fig. 2*F*), respectively, for FGG3X compared to WT mice. These data demonstrate a role for fibrin γ-chain cross-linking in providing firmness to the whole blood clot in the presence and absence of platelets and are in agreement with our previous findings of a decreased Young’s modulus for clots made with recombinant human γQ398N/Q399N/K406R fibrinogen (23). When a range of tissue plasminogen activator (tPA) concentration was added to EXTEM (Fig. 2*G*), no difference in clot lysis time (Fig. 2*H*) and other lysis parameters (Fig. 2 *I*–*K*) were observed between FGG3X and WT mice, indicating no role for fibrin γ-chain cross-linking in determining whole–blood clot lysis efficiency.

**Fig. 3.**
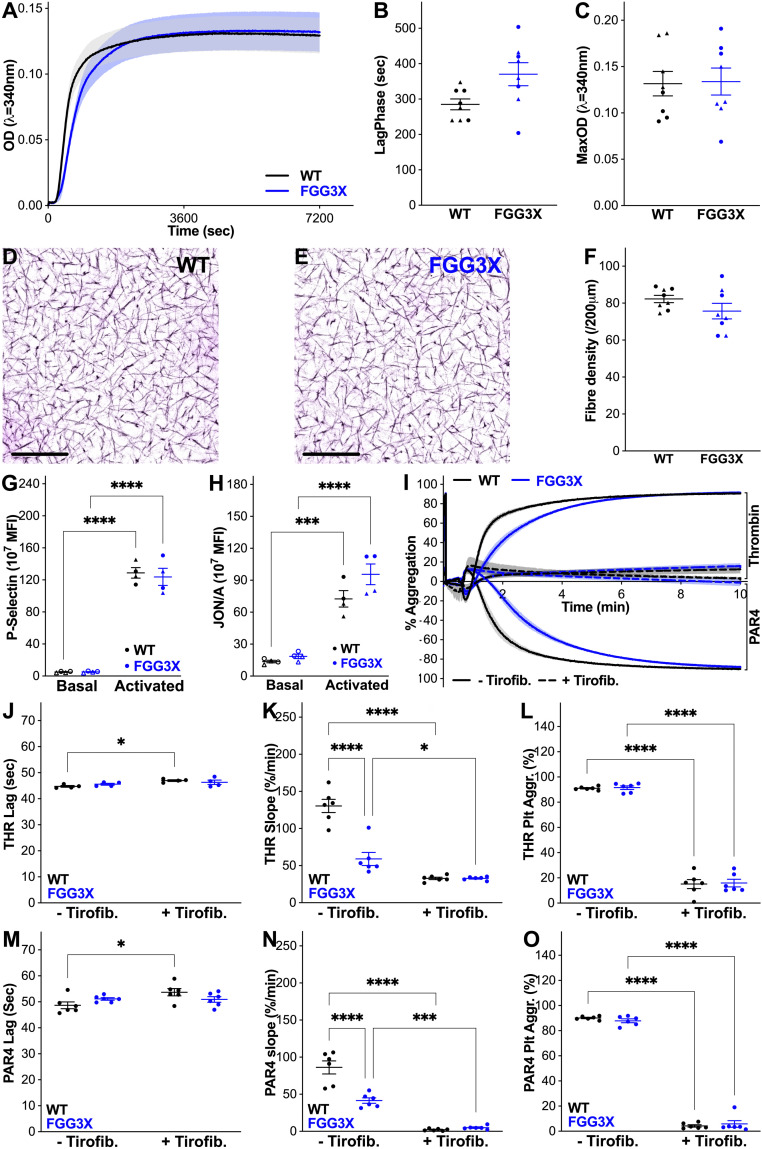
FGG3X mice have similar clot structure and platelet function compared with WT mice. Turbidity analysis of plasma (diluted one-sixth) activated with thrombin and CaCl_2_ (0.1 U/mL and 10 mM) (*A*) showed a small increase in lag phase (*B*) for FGG3X mice but no difference in maximum absorbency (*C*) compared to WT mice. Plasma clot structure (*D* and *E*) and fibrin fiber density (*F*), measured by confocal microscopy (one-eighth plasma, 25μg/mL AlexaFluor^488^-fibrinogen, 0.05 U/mL thrombin, and 10 mM CaCl_2_), were also similar between FGG3X and WT mice (Scale bar, 25 μm). Platelet activation (P-Selectin and α_IIb_β_3_ activation analyzed by JON/A mAb binding induced by 0.1 U/mL thrombin for 20 min) (*G* and *H*) and aggregation (induced by 0.05 U/mL thrombin [THR, *Top*], or 100 µM PAR4 [*Bottom*], over 10 min) (*I*) of FGG3X mice were similar compared to that of WT mice. The rate of aggregation was slightly slower for FGG3X mice at 2 and 3 min (*K* and *N*), but the lag time (*J* and *M*) and final aggregation (*L* and *O*) were identical to that of WT mice. When 5 μg/mL Tirofiban was added (*I*: solid lines without, broken lines with Tirofiban), aggregation was inhibited to a similar extent between FGG3X and WT mice. *n* = 8 (*A–F*), 4 (*G* and *H*), and 6 (*I–O*); [▲] males, [●] females. The data are presented as mean ± SEM and analyzed by Mann–Whitney *U* test (*A–F*) and two-way ANOVA test (*G*–*O*); *P < 0.05, ***P < 0.001, ****P < 0.0001.

Next, we examined whether the effects of FGG3X on clot firmness by ROTEM could have been attributed to changes in clot structure or platelet function. Clots were made with plasma collected from both strains of mice and analyzed by turbidity measurements and confocal microscopy. In vitro, turbidity analysis (Fig. 3*A*) of plasma clots from FGG3X mice showed a 1.3-fold increase in lag phase (Fig. 3*B*), in agreement with our previous publication (24) and ROTEM data, but unchanged maximum absorbency (Fig. 3*C*) compared to WT mice, indicating no major differences in fibrin fibers thickness and density. This was confirmed by confocal microscopy analysis of clots (Fig. 3 *D* and *E*), where the fiber density (Fig. 3*F*) was similar between FGG3X and WT mice. These data indicate that the architecture of clots from FGG3X and WT mice is not different and therefore not responsible for the differences in clot firmness observed by ROTEM. Using flow cytometry, we found that agonist-induced platelet expression of P-selectin (Fig. 3*G*) and activation of α_IIb_β_3_ (Fig. 3*H*) in whole blood were indistinguishable between FGG3X and WT mice. We next examined aggregation using thrombin and also PAR4-specific peptide to account for any potential differences due to the conversion of fibrinogen to fibrin and cross-linking of fibrin by FXIII (Fig. 3*I*). The lag time to aggregation (Fig. 3 *J* and *M*) and overall aggregation response (Fig. 3 *L* and *O*) was unchanged between FGG3X and WT mice, regardless of agonist. We observed a minor reduction in the rate of aggregation for the FGG3X mice up to 3 min, but afterward, the aggregation response was normal (Fig. 3 *I*, *K*, and *N*). Furthermore, the presence of Tirofiban, an inhibitor of α_IIb_β_3_ binding to fibrin(ogen), abolished aggregation in both mouse strains, confirming that fibrinogen-mediated aggregation of FGG3X platelets was largely unaffected (Fig. 3 *I*–*O*). Together, these data show that the reduction in clot firmness by ROTEM in the FGG3X model is attributable to the lack of fibrin γ-chain cross-linking and not to major changes in platelet function or clot architecture.

We then investigated the effect of the lack of fibrin γ-chain cross-linking on whole–blood clot contraction, washed platelet clot retraction, and in vivo clot size in an inferior vena cava stasis model of thrombosis. We found that whole–blood clot contraction kinetics, final clot weight, and final serum hemoglobin content (*SI Appendix*, Fig. S3 *A–D*) were comparable between FGG3X and WT mice, indicating that the lack of γ-chain cross-linking does not affect blood clot contraction, in agreement with previous data showing that red blood cell retention is dependent on α-chain cross-linking and not γ-chain cross-linking (26). The ability of washed platelet clots to retract was also not affected by the loss of fibrin γ-chain cross-linking (*SI Appendix*, Fig. S3 *E* and *F*). These data were further supported by inferior *vena cava* ligation experiments (*SI Appendix*, Fig. S3 *G* and *H*), which showed similar clot weight after 24 h between FGG3X and WT mice, thus indicating that the lack of γ-chain cross-linking does not affect overall clot formation or size in vivo.

### FGG3X Mice Form Clots that Are Less Stable.

**Fig. 4.**
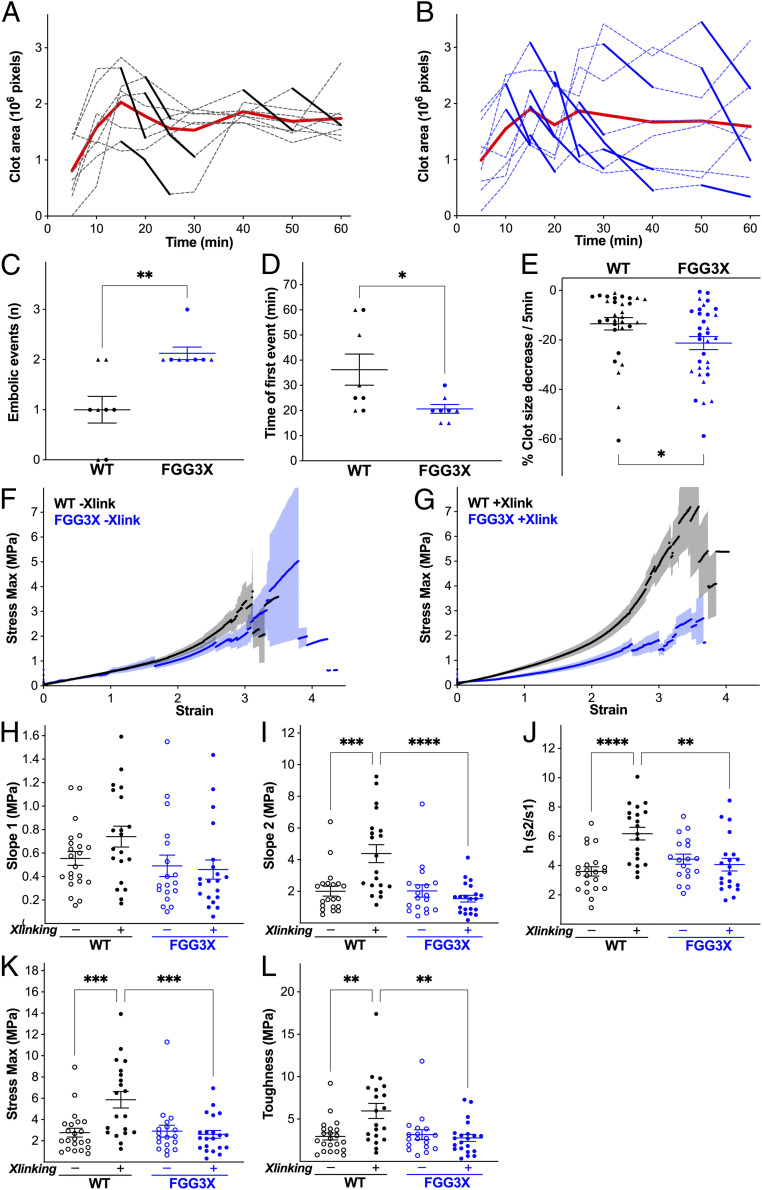
Clot stability and fiber resistance to rupture are reduced in FGG3X mice. Following injection of 100 μg AlexaFluor^488^-fibrinogen and injury to the femoral vein with 10% FeCl_3_ for 3 min, clot size was measured over time by intravital fluorescence microscopy in WT (*A*) and FGG3X (*B*) mice, with the red line representing the average clot area and bold lines representing clot size decreases greater than 25%/5 min (embolic event). The number of embolic events (*C*) was significantly increased, the time for the first embolic event to occur (*D*) was significantly lower, while the overall size of the emboli (*E*) were significantly larger in FGG3X mice compared to WT mice. Fibrin fiber pulling characteristics were measured by lateral atomic force microscopy using purified FGG3X and WT fibrinogens in the absence (*F*) or presence (*G*) of FXIIIa. Low strain stiffness (*H*) was slightly increased, while maximal stiffness prior to rupture (*I*), strain stiffening (*J*), maximum stress before rupture (*K*), and fiber toughness (*L*) were significantly increased by cross-linking in WT but not FGG3X fibrin. *n* = 8 (*A–E*) and 18 to 22 (*F*–*L*); [▲] males, [●] females. The data are presented as mean ± SEM and analyzed by Mann–Whitney *U* test (*C*), χ^2^ test (*D*), Kolmogorov–Smirnov test (*E*), and one-way ANOVA test (*H–L*); *P < 0.05, **P < 0.01, ***P < 0.001, ****P < 0.0001.

A previously described intravital microscopy model of venous thrombosis (27) using FeCl_3_ application to the femoral vein was used to study in vivo clot formation. In this model, thrombus formation in the femoral vein was followed over time (*SI Appendix*, Fig. S4), with measurements of fibrin fluorescence, hence clot size, every 5 to 10 min over the first hour in the FGG3X (Fig. 4*A*) and WT (Fig. 4*B*) mice. Despite the average clot area for each time point being similar between both strains, analyses of the individual kinetic data were consistent, with FGG3X mice demonstrating more frequent drops in clot size between subsequent time points compared with WT mice. When this was quantified, with drops in clot size greater than 25% between time points counted as embolic events (Fig. 4*C*), FGG3X mice showed a significant 2.1-fold (*P* < 0.01) increase in embolic events compared to WT mice. The time for the first embolic event to occur (Fig. 4*D*) was significantly shorter (1.8-fold, *P* < 0.05) in FGG3X mice. The percentage of clot area loss per 5 min was also quantified for all time points (Fig. 4*E*) and showed a significant 1.5-fold increase (*P* < 0.05) in the overall amount of clot embolism in FGG3X mice compared to WT mice. These data suggest that the lack of fibrin γ-chain cross-linking renders the clot more prone to release fragments (emboli) during clot formation.

We next examined fibrin fiber mechanical behavior using lateral atomic force microscopy, to probe fibers made with fibrinogen purified from FGG3X and WT mice. Individual fibers were pulled using a lateral force–sensing atomic force microscope until rupture, and the resulting stress–strain curves (Fig. 4 *F* and *G*) were analyzed. In the absence of cross-linking by FXIIIa, stiffness at low (slope 1, Fig. 4*H*) and high (slope 2, Fig. 4*I*) strains, strain stiffening (s2/s1, Fig. 4*J*), maximum stress before rupture (Fig. 4*K*), and toughness (amount of energy absorbed prior to rupture; Fig. 4*L*) were all similar between both types of fibrinogen. However, after cross-linking by FXIIIa, while initial stiffness was slightly increased (1.3-fold), there was significant increase for large strain stiffness (2.2-fold, *P* < 0.001), strain stiffening (1.7-fold, *P* < 0.0001), maximum stress before rupture (2.1-fold, *P* < 0.001), and toughness (2.0-fold, *P* < 0.01) in WT but not FGG3X fibers. Therefore, cross-linked FGG3X fibers were less stiff before rupture (35%, *P* < 0001), exhibited reduced strain stiffening (75%, *P* < 0.01), ruptured at a lower stress (45%, *P* < 0.001), and had lower toughness (47%, *P* < 0.01) compared to WT fibers, indicating that the lack of γ-chain cross-linking by FXIII renders the FGG3X fibrin fibers more prone to rupture at lower stress relative to WT.

### Thromboembolism Models Show Increased Embolism in FGG3X Mice.

**Fig. 5.**
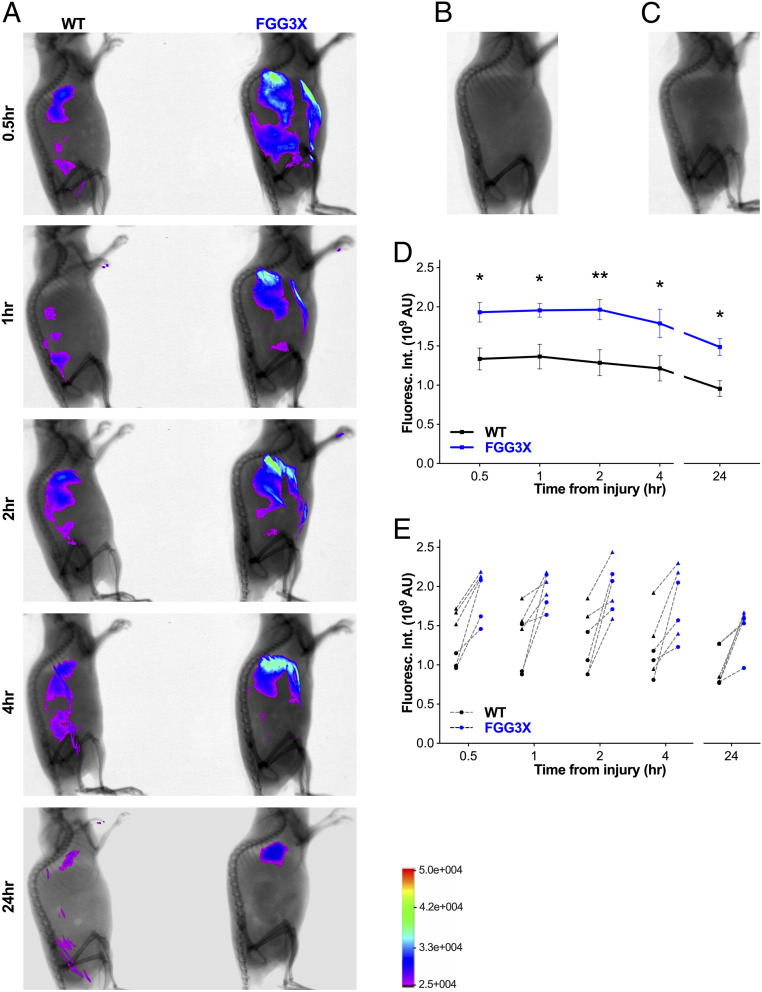
Optical imaging analysis of PE in FGG3X and WT mice. Following injection of 100 μg AlexaFluor^480^-fibrinogen and injury to the inferior vena cava with 5%FeCl_3_ for 3 min, Xtreme (optical + X-ray) imaging of the whole animal was performed at 0.5, 1, 2, 4, and 24 h (*A*), pairing WT (*Left*) and FGG3X (*Right*) mice by body weight (one representative pair shown). Injection of AF^680^-fibrinogen alone (*B*) or application of FeCl_3_ in the absence of AF^680^-fibrinogen (*C*) did not induce any fluorescence signal (2 h timepoint). Lungs total fluorescence intensity was measured for each time point and showed a significant increase of PE in FGG3X mice compared to WT mice (*D*). For each pair, at each time point, FGG3X mice showed a higher fluorescence intensity than WT mice (*E*). *n* = 6; [▲] males, [●] females. The data are presented as mean ± SEM and analyzed by two-way ANOVA test; *P < 0.05, **P < 0.01.

In order to investigate the effects of γ-chain cross-linking by FXIIIa on clot stability and embolism in a pathophysiological setting, we developed two protocols to specifically evaluate the level of thromboembolism to the lungs (PE) from thrombi in the inferior vena cava. First, we used optical imaging coupled to X-ray to observe live appearance of emboli into the lungs of mice undergoing inferior vena cava injury using FeCl_3_ (*SI Appendix*, Fig. S5), after prior injection of fluorescent fibrinogen into the mice. The whole-body fluorescence levels in FGG3X and WT mice were observed 0.5, 1, 2, 4, and 24 h postinjury (Fig. 5*A*). We observed that fluorescence accumulated specifically in the lungs over time after inferior vena cava thrombosis due to clot embolism. In contrast, fibrin accumulation was not observed elsewhere in the circulation of the mice. Control experiments with only fluorophore injected (Fig. 5*B*) or FeCl_3_ injury without fluorophore present (Fig. 5*C*) showed no signal in these mice 2 h postinjury. Quantification of total fluorescence in the lungs showed that 30 min postinjury, FGG3X mice showed a significant 1.4-fold increase (*P* < 0.05) in clot emboli compared to WT mice (Fig. 5*D*). This increase in fluorescence intensity was also significantly higher in FGG3X mice at 1 (1.4-fold, *P* < 0.05), 2 (1.5-fold, *P* < 0.01), 4 (1.5-fold, *P* < 0.05), and 24 h (1.6-fold, *P* < 0.05), compared to WT (Fig. 5*D*). Imaging was performed in pairs of matching gender and weight. For each pair, FGG3X mice showed higher fluorescence intensity in the lungs than their WT counterparts at every time point (Fig. 5*E*).

**Fig. 6.**
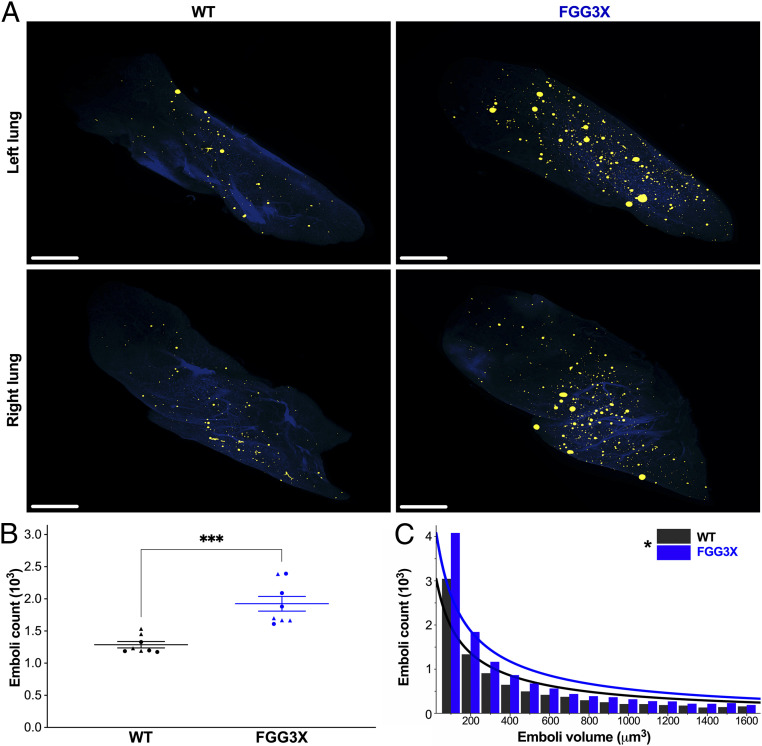
Light sheet microscopy of clot emboli in the lungs of FGG3X and WT mice. Following injection of 100 μg AlexaFluor^647^-fibrinogen and injury to the inferior vena cava with 5% FeCl_3_ for 3 min, perfusion fixation of the mice with PFA after 57 min, and injection of FITC-albumin in the circulation prior collection and clearing of the lungs, light sheet fluorescence microscopy imaging of the lungs (*A*) showed that the total emboli count per mouse (*B*) was significantly increased in FGG3X mice. The distribution of all the emboli volumes (*C*) showed that FGG3X mice produced significantly more emboli, irrespective of their size, than WT mice (Scale bar, 1 mm). *n* = 8; [▲] males, [●] females. The data are presented as mean ± SEM and analyzed by Mann–Whitney *U* test (*B*) and Kolmogorov–Smirnov test (*C*); *P < 0.05, ***P < 0.001.

Next, we used light sheet microscopy to image and quantify clot emboli in the lungs of mice where the inferior vena cava was injured with FeCl_3_ following tail vein injection of fluorescent fibrinogen to visualize clots. Mice underwent whole-body fixation and perfusion with fluorescent albumin (in gelatin) 1 h postsurgery in order to visualize the vasculature. Lungs were imaged by light sheet microscopy, and three-dimensional (3D) fluorescence reconstructions of organs were created using IMARIS (Fig. 6*A*). In this model, FGG3X mice showed a significant 1.5-fold increase (*P* < 0.001) in pulmonary emboli count compared to WT (Fig. 6*B*). The distribution of the emboli size (Fig. 6*C*) was also significantly different (*P* < 0.05) between both strains, since FGG3X mice exhibited a higher number of pulmonary emboli for each volume range compared to WT mice. Together, these data demonstrate that the lack of fibrin γ-chain cross-linking increases embolism in the venous circulation, leading to an increased number and volume of pulmonary thromboembolic events.

## Discussion

We describe a murine model of fibrinogen, FGG3X, in which all three conserved fibrin γ-chain cross-linking sites have been mutated, leading to a total lack of γ-chain cross-linking by FXIIIa. While blood parameters were otherwise identical to that of the background C57BL/6 strain, and fibrin clot structure and platelet physiology remained largely unchanged, ex vivo whole–blood clot firmness was decreased in FGG3X mice due to the absence of γ-chain cross-linking. We next developed specific protocols to study thromboembolism using powerful X-ray and fluorescence imaging combined with light sheet microscopy. We found that during in vivo thrombus formation using an inferior vena cava thromboembolism model, FGG3X produced clots with reduced stability, which resulted in a larger number of pulmonary embolic events. These data indicate that fibrin γ-chain cross-linking is critical to thrombus stability in vivo and reduces clot fragmentation, thereby reducing life-threatening thromboembolic disease. Future treatment options should be developed to exploit this mechanism for the treatment and prevention of fatal thromboembolic disease by specifically targeting fibrin α-chain cross-linking–dependent erythrocyte retention, therefore clot size and burden, while allowing for normal FXIIIa-mediated fibrin γ-chain cross-linking to prevent embolism.

Clinically, treatment of thrombosis is largely based on anticoagulation, with little attention for the quality or stability of the blood clot. Clot quality and stability, however, have been reported to play a role in thromboembolism (9–12), an important and dangerous sequela of thrombosis, leading to PE. As such, it is important to fully understand the role of fibrin cross-linking in the formation and stability of thrombi and their subsequent embolization. On the basis of these considerations, we consider that a more-complete understanding of the implications of cross-linking by FXIIIa on clot stability and embolism is urgently needed in order to optimize future treatment regimens for thrombosis and thromboembolism.

We previously generated a recombinant human fibrinogen variant γ-3X (23), in which all the three γ-chain cross-linking sites were mutated (γQ398N/Q399N/K406R). This fibrinogen allowed for careful and detailed dissection of the specific roles of α–α and γ–γ cross-links in clot structure and mechanical properties in vitro (23–25). Injection of human fibrinogen γ-3X mutant in fibrinogen^−/−^ mice demonstrated that fibrin α-chain plays a central role in red blood cell retention during whole–blood clot contraction and is therefore a key determinant of clot and thrombus size (26). Since the importance of fibrin γ-chain cross-linking in clot stability has not yet been studied in vivo due to a lack of an appropriate animal model, and since the γ-chain cross-linking sites are highly conserved in human and mouse fibrinogens, we generated a FGG3X genetically modified murine model in order to investigate the relevance of γ-chain cross-linking in vivo. Except for a total lack of fibrin γ-chain cross-linking, the basal phenotype of these mice was similar to WT, and (re)bleeding was not affected, indicating that primary hemostasis and α-chain cross-linking within the subsequent fibrin clot are sufficient to prevent bleeding. We confirmed in this murine model previous in vitro human data showing that a lack of fibrin γ-chain cross-linking does not impair red blood cell retention during clot contraction (26). Our results differ from that of FXIII-A–deficient human (28) and mouse (29) clot retraction assays, where cross-linking of both fibrin α- and γ-chain is fully abolished by the absence of FXIII activity, resulting in impaired clot retraction. We found that the presence of normal fibrin α-chain cross-linking in the FGG3X mice maintains the whole–blood clot contraction phenotype observed in WT mice. These observations were confirmed by inferior vena cava stasis experiments, showing that in a static in vivo environment (where clot embolization is not possible due to complete vessel ligation), the lack of γ-chain cross-linking does not impact on the formation and size of venous thrombi. Additionally, the mutations of γ-Q398N, γ-Q399N, and γ-K406R residues, located near the α_IIb_β_3_ binding site (γ-404 to 411 in mouse and human) (30), did not affect washed platelets activation, aggregation, and clot retraction. A study by Jiroušková et al. (31) showed that an antibody (7E9) directed against the fibrin γ-chain C terminus, which sterically hinders fibrinogen–α_IIb_β_3_ interaction and fibrin cross-linking by FXIIIa, resulted in reduced platelet aggregation and clot contraction. Additionally, in an arterial thrombosis model, γ-Δ5 mice (which lack the N-terminal residues of the γ-chain responsible for α_IIb_β_3_ interaction) and WT mice administered with the 7E9 antibody formed smaller thrombi with limited embolization. Our data show that the conservative Q-N and K-R mutations in the three cross-linking sites for FXIIIa that are close to integrin binding site do not influence platelet binding nor clot contraction, yet γ-chain cross-linking is completely abolished. Importantly, in vivo experiments using the FGG3X mice and intravital microscopy imaging on clots formed in the femoral vein following application of FeCl_3_ (27) showed that these mice form clots that are less stable, most likely due to the reduced toughness (increase rupture potential) observed by single-fiber pulling experiments using lateral atomic force microscopy, resulting in an increased number of embolic events in the early stages of clot formation.

Next, two inferior vena cava thrombosis and PE protocols were developed, showing a critical role for fibrin γ-chain cross-linking in preventing clot embolism in the venous circulation. Our model and protocol show that a loss of fibrin γ-chain cross-linking and increased susceptibility to fiber rupture correlated with the formation of less-stable venous thrombi that were more prone to fragmentation and subsequent embolization to the lungs. We found that FGG3X mice produced a higher degree of PE than WT mice at all time points observed. At 2 h post–vena cava thrombosis, the fluorescence intensity in the lungs of both FGG3X and WT mice decreased at a similar rate, most likely due to fibrinolysis of the emboli taking place. The rate of lysis was similar between both strains, in agreement with our ex vivo ROTEM data showing that clot lysis was not affected by the lack of fibrin γ-chain cross-linking. With clot lysis being unaffected, our data instead indicate that clots without fibrin γ-chain cross-linking are more readily deformed under shear stress, with dissociation of knob-hole and other noncovalent bonds, which are not as strong as covalent bonds (e.g., γ-chain cross-links present in WT but not FGG3X fibrin) and hence rupture of individual fibers, which render the clots more prone to fragmentation and embolism.

**Fig. 7.**
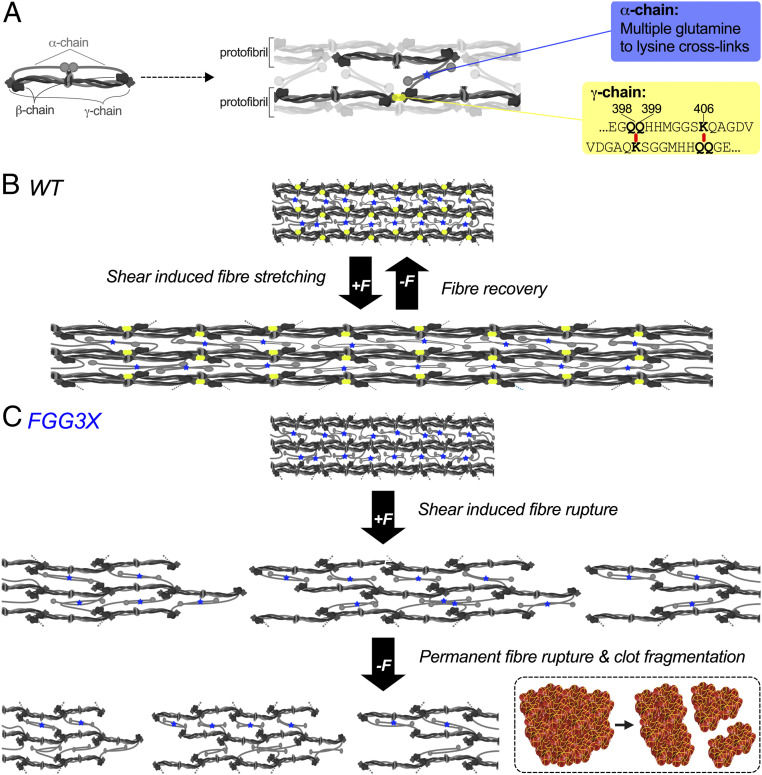
Fibrin γ-chain cross-linking increases clot stability and reduces embolism. Fibrinogen is a soluble heterotetrametric molecule, consisting of two α-, two β-, and two γ-chains (*A*, *Left*), which is proteolytically converted by thrombin into insoluble fibrin. Fibrin monomers polymerize in a half-staggered arrangement to form protofibrils which interact laterally via α-chains to form fibers (*A*, *Middle*). FXIIIa stabilizes the clots by forming longitudinal intraprotofibril cross-links between glutamine 398 or 399 and lysine 406 (398/399 and 406 in human) of adjacent γ-chains, as well as lateral interprotofibril cross-links between multiple glutamine and lysine residues of adjacent α-chains (*A*, *Right*). In normal conditions (*B*, WT mice), blood flow induces strain (F) on the fibrin fibers, resulting in deformation of the clots which is reduced by γ-chain (intraprotofibril, yellow rectangles) and α-chain (intra- and interprotofibrils, blue stars) cross-linking. Clots from FGG3X mice (*C*) exhibit a lack of γ-chain cross-linking, resulting in dissociation of the D–E–D interactions, slippage of the fibrin molecules along the protofibrils, and permanent deformation and rupture of protofibrils and hence individual fibers. While fibrin α-chain cross-linking partially rescues the integrity of the clots, the compromised γ-chain cross-linking predisposes to clot fragmentation and release of emboli (*Insert*) in places that lack (randomly orientated) interprotofibril α-chain cross-links (images were created with BioRender.com).

A paradigm is proposed in which in the absence of γ-chain cross-linking, the force of flow applied to fibrin protofibrils likely leads to the dissociation of the D–E–D interactions, slippage of the fibrin molecules along the protofibrils, fiber rupture, and permanent clot deformation (Fig. 7). Fibrin α-chain cross-linking may in part rescue the integrity of the clots via intra- and interprotofibril cross-links. However, in places where those interactions do not occur, due to the random nature of the α-chain cross-linking orientation, rupture of the fibers results in subsequent clot embolism. This phenomenon will be less common in clots from WT mice, as fiber rupture and clot fragmentation are prevented by the presence of fibrin γ-chain cross-links. This model is further supported by a previous study by Liu et al. on in vitro fibrin mechanical properties, which concluded that “fast-forming γ–γ cross-links along the axis may enhance elasticity and prevent rupture of the (nascent) fibers” (17). Our data indicate that this is also the case in vivo using protocols to study thromboembolism, suggesting a key role for γ-chain cross-linking in the pathophysiology of this disease. These findings are further supported by a recent clinical study showing that plasma samples from patients with recurrent VTE have reduced elastic modulus compared to samples from patients with nonrecurrent VTE (12), indicating that optimal clot elasticity is critical to prevent VTE recurrence.

Prior in vivo studies investigating thromboembolism involved injection of exogenous clots or thrombotic substance in the venous system (e.g., thrombin or collagen/epinephrine) with the use of histology to detect the emboli within the lungs (32). Shaya et al. recently applied FeCl_3_ to the femoral vein to study thrombus formation and performed histology of the lungs to analyze the effect of dabigatran (a direct thrombin inhibitor) on PE (33). Interestingly, these authors found that dabigatran decreased clot stability and increased PE in this model, which they attributed to reduced FXIII cross-linking activity since untreated FXIII^−/−^ mice showed similar rates of PE as WT mice treated with dabigatran. We chose FeCl_3_ as trigger for thrombosis due to its reproducibility in terms of the size of the ensuing thrombus. Furthermore, our aim was to study clot stability and embolism rather than the development of the clot itself, justifying the use of FeCl_3_ as a suitable model to test our hypothesis. However, future studies may combine whole-body/organ emboli imaging described here with other models of venous thrombosis such as inferior vena cava ligation and stenosis models, which have also been shown to lead to embolization (34).

We developed and characterized methodological approaches to study thromboembolism. We used live in vivo optical imaging coupled with X-ray to analyze thromboembolism in real time in living mice. Advantages of this method are that the fluorescently labeled fibrin is stable enough to allow measurements for at least 24 h and that the method can be applied to other models of thrombosis which tolerate animal recovery. Limitations are that intensity measurements do not provide detailed information on the number and volume of the emboli generated or their precise location within the lungs and that imaging is only possible in those parts of the body that are not masked by bones (e.g., the lungs but not the brain) or very opaque tissues. Information on emboli to organs covered by bone, however, can be provided by the second, complementary imaging method that we used, involving terminal light sheet microscopy following perfusion fixation and tissue clearing. The light sheet microscopy data 1 h postinjury were in perfect agreement with the 1-h time point from the optical imaging experiments, showing that FGG3X mice demonstrate an increased number of pulmonary emboli and that for each volume bracket, FGG3X produced more emboli, which should result in an overall higher fluorescence intensity. Advantages of this technique are the detailed quantification of emboli numbers and volumes and accurate location of these emboli in the organ as well as the imaging of any organ in the body, regardless of its location. Limitations are that this is a terminal procedure, therefore requiring a larger number of animals if more than one time point is required. Thus, the combination of both light sheet microscopy and whole-body combined optical and X-ray imaging provides powerful and comprehensive analysis of thromboembolic disease. Furthermore, it may be possible to combine both protocols in sequence, performing optical imaging first, followed by light sheet imaging at the terminal endpoint. This would require that the type of injury allows for recovery and that fluorophores and filters are compatible with both approaches.

Previous clot contraction assays and our current in vivo thromboembolization data provide strong evidence that fibrin α- and γ-chain cross-linking play complementary roles in venous thrombosis via different mechanisms. Byrnes et al. showed that fibrin α-chain (but not γ-chain) cross-linking plays an important role in red blood cell retention within the clot (26), thereby increasing the size of venous thrombi. Our study demonstrates that γ-chain cross-linking is critical for the stability of venous thrombi, therefore reducing breakdown and embolism. While anticoagulation is currently used as treatment for thromboembolic diseases, any possible effects of this treatment on embolism have so far been largely ignored. Based on our current data, and in agreement with Byrnes et al. (26), we propose that specific inhibitors of fibrin α-chain cross-linking should be developed to reduce red blood cell content within thrombi, therefore size and burden of venous thrombi. Moreover, specific α-chain cross-linking inhibitors would not impact on the critical fibrin γ-chain cross-linking by FXIIIa, thereby preventing thromboembolic effects as shown in our current study.

In conclusion, we present the development and characterization of a genetically modified murine model in which the residues responsible for fibrin γ-chain cross-linking are mutated. We also developed and characterized protocols to study thromboembolism using powerful imaging methods. These models demonstrate the essential importance of γ-chain cross-linking in clot viscoelastic properties. We show a critical role of fibrin γ-chain cross-linking by FXIIIa in stabilizing clots and reducing thromboembolic events in the venous (PE) circulation in vivo. These data demonstrate important mechanisms related to clot mechanical properties in thromboembolic disease, indicating a key future target for therapeutic intervention and prevention of this leading cause of death that remains poorly treated to date.

## Methods

### Ethics.

All procedures were approved by the University of Leeds and the University of Sheffield Ethics Committees and performed under project license numbers 70/8115 and P144DD0D6 (held by Stephen Wheatcroft at Leeds University) and 70/8532 (held by Victoria Ridger, University of Sheffield) according to the Home Office Animals (Scientific Procedures) Act 1986. Both males and females were used, aged 6 to 8 wk at the time of the experiments.

### Materials.

Recombinant murine thrombin (Haematologic Technologies Inc) was reconstituted to 250 U/mL in ddH_2_O and stored at −80 °C. Recombinant human tPA (Pathway Diagnostics) was diluted in Tris-buffered saline (TBS; 0.05 M Tris HCl, 0.1 M NaCl, pH 7.4) to 1,400 nM and stored at −80 °C. EZ-link pentylamine-biotin (Thermo Fisher Scientific) was diluted in ddH_2_O to 30 μM and stored at −20 °C. AlexaFluor^488^, AlexaFluor^647^, and AlexaFluor^680^ protein labeling kits were purchased from Thermo Fisher Scientific. All other chemicals were obtained from Sigma unless stated otherwise.

### Generation and Maintenance of FGG3X Mice.

A genetically modified murine FGG3X line, in which the fibrinogen γ-chain cross-linking sites have been mutated, were generated by GenOway as follows: Human residues γ-Q398, γ-Q399, and γ-K406 (mature sequence) are conserved in murine fibrinogen (γ-Q398, γ-Q399, and γ-K406; *SI Appendix*, Fig. S1), with the corresponding codons located in exon 9 of the murine *Fgg* gene. Homologous recombination in embryonic stem (ES) cells was performed using a targeting-vector–containing region homologous to the murine genomic *Fgg* sequences. The targeting vector (ARI1-HR) was generated by cloning the mouse genomic DNA encompassing the murine *Fgg* gene regions surrounding the target exon 9 (exon 7 to 5′-UTR [untranslated region]) into the targeting vector, inducing the three point mutations (Q398N, Q399N, and K406R), and inserting a neomycin selection cassette (Neo) (*SI Appendix*, Fig. S2). The vector was linearized and transfected into ES cells by electroporation, and cells were selected for resistance to neomycin, before the correct recombination events were validated by PCR and Southern blot. The ES cells containing the correct recombination were then injected into blastocysts (3.5-d-old embryos), which were subsequently implanted into C57BL/6 pseudopregnant females, resulting in the generation of chimeric mice. Male mice with chimerism rate above 50% were mated with C57BL/6-Cre females to generate heterozygous mice carrying the Neo-excised knock-in allele, which were bred for the generation of homozygous mice. Upon transfer to the University of Leeds, FGG3X mice were backcrossed onto our C57BL/6J strain over 10 generations and maintained in individually ventilated cages at 21 °C, 50 to 70% humidity. The light/dark cycle was 12/12 h, and mice were fed on standard chow diet ad-libitum. Experimental units were one mouse per cage.

### Mouse Growth and Blood Sampling.

WT and FGG3X mice were weighed once a week, at the same time of the day from 2 to 12 wk old. Experiments were performed with eight mice per group. For blood sampling, animals were bled from the inferior vena cava under terminal anesthesia using 10% vol/vol 0.109 M sodium citrate as anticoagulant. Blood was centrifuged at 14,000 *g* for 10 min at room temperature (RT) for plasma preparation. The platelet-poor plasma supernatant was collected, aliquoted, and stored at −80 °C.

### Bleeding Time and Rebleeding Events.

To measure bleeding time, the distal 3-mm segment of the mouse tail tip was sectioned using a scalpel under anesthesia and immersed in a microtube containing isotonic saline (37 °C) (35). Bleeding time was determined using a stop clock. Measurement of rebleeding events was performed using a method adapted from Molina et al. (36); the mice were returned to individual cages 5 min after cessation of bleeding, and a filter paper was applied to the end of the mouse tail every 15 min over a 1-h period. Appearance of fresh blood on the filter paper was counted as a rebleeding event. Experiments were performed with eight mice per group.

### Fibrinogen Levels and FXIII Activation Rate.

Plasma fibrinogen levels were determined using a murine fibrinogen total antigen enzyme-linked immunosorbent assay (ELISA) Kit (MyBioSource) following manufacturer’s instructions. Measurement of FXIII activation was performed using a modified 5-(biotinamido)pentylamine incorporation assay (27). Nunc-Immuno 96-MicroWell plates were coated with 100 μL 10 μg/mL N,N-dimethylated casein for 40 min at RT and blocked with 300 μL 1% bovine serum albumin (BSA) in TBS (pH 8.3) for 90 min at 37 °C. Plates were washed with 4× 300 μL TBS (pH 8.3) and 10 μL plasma samples (diluted one-tenth in TBS [pH 8.3]) were added to the wells in triplicate. A total of 90 μL activation mix (111 μM dithiothreitol [DTT], 0.3 μM biotinylated pentylamine, 11 mM CaCl_2_, and 2.2 U/mL thrombin) were added and the reactions were stopped at 0, 20, 40, 60, 80, 100, and 120 min by adding 200 μL 200 mM ethylenediaminetetraacetic acid. Plates were washed with 4× 300 μL 0.1% [vol/vol] Tween 20 in TBS (pH 8.3), and 100 μL 2μg/mL streptavidin in 1% [wt/vol] BSA (in TBS-Tween) were added and incubated for 60 min at 37 °C. Following washes with 4× 300 μL TBS-Tween, 100 μL 1 mg/mL phosphatase substrate (in 1 M diethanolamine) were added for 7 min, and the reaction was stopped by adding 100 μL 4 M NaOH. Absorbency was measured at 405 nm using a PowerWave HT Microplate Spectrophotometer (BioTek). The rate of pentylamine incorporation over time was used as an indicator of FXIII activation. Measurements were performed in triplicate, with eight mice per group.

### Fibrinogen Purification.

Fibrinogen was purified from pooled mouse plasma by ammonium and ethanol precipitations, using a protocol adapted from Dietrich et al. (37). All steps were performed at 4 °C. A protease inhibitor mixture (55 mM ε-aminocaproic acid, 55 mM benzamidine, 11 μM pepstatin, 11 μM leupeptin, and 1.1 mM phenylmethylsulfonyl fluoride, in TBS) was added (1:10 vol/vol) to plasma and 1 vol saturated ammonium sulfate (760 g/L) was slowly (drop-by-drop) added to 3 vol plasma and incubated for 2 h. Following centrifugation at 12,000 *g* for 15 min, the pellet was resuspended in 2-(*N*-morpholino) ethanesulfonic acid (MES) buffer (55 mM ε-aminocaproic acid, 55 mM benzamidine, 1.1 μM pepstatin, 1.1 μM leupeptin, 110 μM phenylmethylsulfonyl fluoride, 22 mM 2-(N-morpholino)ethanesulfonic acid, in ddH_2_O, pH 6.6), and the whole process was repeated a second time, followed by pellet resuspension and dialysis in TBS for 1 h. Then, 1 vol ice-cold 100% ethanol was added drop-by-drop to 13 vol ice-cold suspension and incubated on ice for 1 h. Following centrifugation at 12,000 *g* for 15 min, the pellet was resuspended and dialyzed against TBS overnight before the concentration was determined using a ND-100 Spectrophotometer (Thermo Fisher Scientific).

### Fibrin Cross-Linking.

Clotting mixtures (20 μL) containing fibrinogen (0.25 mg/mL), zymogen FXIII (10μg/mL), thrombin (0.5 U/mL), and CaCl_2_ (10 mM) were incubated at 37 °C for 0, 2, 5, 10, 15, 20, 30, 60, and 120 min. Reactions were stopped by adding 4× NuPAGE lithium dodecyl sulphate (LDS) Sample Buffer (ThermoFisher Scientific) and 10× NuPAGE Sample Reducing Agent (Thermo Fisher Scientific), immediately followed by heating at 90 °C for 10 min. Samples and molecular weight marker (Precision Plus Protein Dual Color Standards; BioRad) were run onto a NuPAGE 4 to 12% Bis-Tris Protein Gel (Thermo Fisher Scientific), and gels were stained using InstantBlue (Expedeon). Protein bands were visualized and quantified using Genesys and GeneTools softwares (Syngene). Band quantification for each lane was relative to the amount of B-β chain staining. Experiments were performed in triplicate.

### Fibrinogen Labeling.

For in vivo experiments, fibrinogen purified from FGG3X and WT mice was labeled with AlexaFluor 488, 647, or 680 depending on the filters available for each setup. Labeling was performed following manufacturer’s instructions (Thermo Fisher Scientific).

### Hematological Blood Parameters.

Freshly obtained blood samples were run onto a KX-21N Automated Hematology Analyzer (Sysmex). Discriminator values were changed for leukocyte counts as follows: LD 30 fl, T1 66 fl, and T2 84 fl. Experiments were performed in triplicate with eight mice per group.

### Rotational Thromboelastometry.

Freshly obtained blood samples were run on a ROTEM-Delta (Werfen) for thromboelastic analysis. For clotting analysis, 103 μL blood was mixed with 3 μL TBS, 7 μL EXTEM or FIBTEM, and 7 μL STARTEM (CaCl_2_) reagents before data were acquired using the EXTEM and FIBTEM channels (respectively). For lysis analysis, 107 μL blood was mixed with 7 μL EXTEM and 7 μL STARTEM reagents and 3 μL tPA (final concentration range 2.5 to 100 nM) before data were acquired using the EXTEM channel. Measurements were performed with eight mice per group.

### Fibrin Polymerization and Clot Structure.

Polymerization of fibrin was studied by turbidity analysis as previously described (24). A total of 25 μL plasma (one-third) were transferred into 384-well plates in triplicate. A total of 25 μL CaCl_2_ and thrombin mix (0.1 U/mL and 10 mM final reaction concentrations) were added to initiate clotting, and absorbency was measured at 340 nm every 12 s for 2 h at 32 °C using a PowerWave HT Microplate Spectrophotometer (BioTek). Clot structure was analyzed by laser-scanning confocal microscopy, as previously described (24). Plasma, AlexaFluor^488^-fibrinogen, CaCl_2_, and thrombin (one-eighth, 25 μg/mL, 10 mM, and 0.05 U/mL final concentrations, respectively) were mixed, transferred into the channel of an uncoated Ibidi μ-slide VI (Thistle Scientific), and incubated in a dark humidity chamber for 60 min. Clots were imaged using an inverted Zeiss LSM-880 microscope (Carl Zeiss) with a 40× oil immersion objective lens. Optical z-stacks were obtained every 0.5 μm over 10 μm and combined into single projected images. Fiber density was determined by counting the number of fibers crossing 10 arbitrary lines of fixed length (200 μm) drawn through a single optical section. Experiments were performed in triplicate with eight mice per group.

### Platelet Functions.

Platelet activation was measured in whole blood, supplemented with 10 μM Gly-Pro-Arg-Pro-NH_2_ and fluorescein isothiocyanate (FITC)-conjugated anti–P-selectin (BD Biosciences) or PE-conjugated JON/A (anti-α_IIb_β_3_; Emfret) antibodies, incubated with or without thrombin (0.1 U/mL) for 20 min at 37 °C (38). Whole blood was subsequently fixed with 1% paraformaldehyde (PFA) (in phosphate-buffered saline [PBS]) for 10 min and analyzed by fluorescence-activated cell sorting using a CytoFLEX Flow Cytometer (Beckman Coulter). A compensation matrix was applied, and analysis was performed with the CytExpert software version 2.1 (Beckman Coulter). Experiments were performed in four mice per group.

Platelet aggregation was measured using suspended washed platelets. Platelet-rich plasma was obtained by centrifugation of whole blood supplemented with 200 µL modified Tyrode’s buffer (MTB; 150 mM NaCl, 5 mM Hepes, 0.55 mM NaH_2_PO_4_, 7 mM NaHCO_3_, 2.7 mM KCl, 0.5 mM MgCl_2_, 5.6 mM d-glucose, pH 7.4) at 100 *g* for 5 min at RT. The resulting pellet was resuspended in MTB and recentrifuged at 1,000 *g* in the presence of PGI_2_ (200 nM) for 6 min, the washed platelet pellet was resuspended in MTB, and counts were adjusted to 2.5 × 10^8^ plt/mL using a Z1 Coulter Particle Counter (Beckman Coulter). Platelet aggregation was performed using 250 μL washed platelets at 2 × 10^8^ plt/mL, calibrated against MTB. Platelets were stimulated with thrombin (0.05 U/mL) or PAR4 (100 µM) with or without the pretreatment of Tirofiban (5 μg/mL), and aggregation was recorded under constant stirring conditions (1,000 rpm) for 10 min at 37 °C using an AggRAM aggregometer (Helena Biosciences Europe). Experiments were performed in six mice per group.

### Intravital Microscopy.

In vivo visualization of clot formation was performed as previously described (27). Mice (6- to 8-wk-old) were anesthetized by intraperitoneal injection of ketamine/atropine/xylazine. The carotid artery was cannulated to allow for maintenance of anesthesia and injection of 100 μg AlexaFluor^488^-fibrinogen 5 min prior to exposure of the femoral vein and application of a 10% [vol/vol] FeCl_3_-saturated filter paper (3 × 2 mm) for 3 min. Real-time observation of clot formation started 2 min after removal of the FeCl_3_ filter paper and washing with isotonic saline using an upright Nikon Eclipse E600-FN microscope (Nikon) equipped for fluorescence microscopy with a water-immersion 40/0.80-W objective. The green channel (488 nm) was recorded using Slidebook Imaging Software version 5.0 (Intelligent Imaging Innovation). Clot size for each time point was determined as a combination of area and intensity of green pixels. Experiments were performed with eight mice per group.

### Lateral Atomic Force Microscopy.

The mechanical response of individual fibrin fibers upon lateral stretching was measured as described previously (17). Briefly, clots were made on a striated surface (25, 39), with resulting fibers forming over the trenches. Individual fibers were pulled laterally with the atomic force microscope (AFM) cantilever, and the lateral deflection of the cantilever was registered and used to calculate the force and the stress on the fiber. Details are provided in *SI Appendix, Methods*.

### Optical and X-ray Imaging.

Mice (8-wk-old) were anesthetized with 1.5% isoflurane (Piramal Critical Care) in oxygen (2 l/min flow rate). 100 μg of AlexaFluor^680^-fibrinogen per 10 g of mouse body weight were injected into the tail vein, and the abdomen, chest, and sides of the mice were shaved to allow for imaging. The inferior vena cava was exposed after midline laparotomy and exteriorization of the bowels and separated from the abdominal aorta using a plastic spacer (40). A filter paper (2 × 3 mm) soaked with 5% FeCl_3_ was applied onto the isolated vessel for 3 min before removing the filter paper and washing the abdominal cavity with saline. The bowels were moved back into the abdominal cavity with saline, and the muscle and skin layers were sutured sequentially. Animals were immediately injected with 100 μL 0.1 mg/mL Vetergesic (Ceva Animal Health Ltd) for pain relief. Using the in vivo Xtreme II optical imaging system (Bruker), fluorescence (excitation 680 nm, emission 700 nm) and X-ray in whole mice was imaged under anesthesia at 0.5, 1, and 2 h postinjury, before animals were allowed to recover, and then at 4 and 24 h postinjury. Animals were imaged in three positions (frontal, sagittal left, and sagittal right) for each time point. Using Molecular Imaging software version 7.5.2.22464 (Bruker) and X-ray images, the lungs were delineated using the “ROI free form” tool (*SI Appendix*, Fig. S5), and fluorescence intensity was quantified for each plane. The values for each image plane were added, determining the total fluorescence intensity. Each set of experiments comprised of one FGG3X and one WT mouse in pairs, determined by gender and weight. Experiments were performed with six mice per group.

### Light Sheet Microscopy.

Mice (6- to 8-wk-old) were anesthetized by intraperitoneal injection of ketamine/atropine/xylazine. AlexaFluor^647^-fibrinogen (100 μL per 10 g of body weight) was injected into the tail vein. The inferior vena cava was isolated over a plastic sheet spacer (40), and a 2.5% FeCl_3_-soaked filter paper (2 × 3 mm) was applied for 3 min. Remaining FeCl_3_ was washed off with isotonic saline, and the mice were kept in the dark for a further 57 min. The mice were then slowly perfused with 20 mL PBS (+50 U/mL heparin), 15 mL 4% PFA, and 10 mL hydrogel (41) (0.8 mg/mL FITC-Albumin, 2% wt/vol gelatin, in PBS). Mice were immediately placed on ice for at least 30 min before the lungs were harvested and transferred to 4% PFA overnight, at 4 °C, in the dark. All subsequent steps were performed in the dark. Next day, the lungs were dehydrated in 20, 40, 60, 80, and 100% methanol solutions (in ddH_2_O) for 1 h each, shaking at 200 rpm at RT, before being left overnight in fresh methanol solution. The left and right lungs were then surgically separated before being optically cleared by incubation in 66% dichloromethane (DCM)/34% methanol solution for 3 h at RT, then twice in 100% DCM for 15 min, shaking at 200 rpm. Finally, the lungs were transferred into benzyl-ether (DBE) for at least for 72 h until imaging (42). The lungs were imaged using a LaVision Ultramicroscope II light sheet microscope coupled with a 0.63× MV PLAPO 2XC objective (Olympus) with lens protector, and samples were immersed in an imaging chamber filled with ethyl cinnamate (same refractive index as DBE). Samples excitation was performed with 470- and 630-nm lasers, producing a 5-µm-thick light sheet, with emitted light collected using 525/550- and 680/630-nm filters, respectively. Two separate image *z*-stacks for the green (hydrogel in blood vessels) and red (emboli) channels were generated using Imspector Pro software version 5.1.328 (Lavision Biotec GmbH). Analysis of the datasets was performed using IMARIS software versin 9.3.0 (Oxford Instruments). The whole image stack was used to create a 3D image reconstruction using identical image parameters for all samples. Volumetric analysis of the emboli was performed using the “volume” tool, with the smallest volume cutoff of 50 μm^3^ (based on smallest capillaries being around 4 to 5 μm in diameter). The volume of individual emboli and the total number of emboli were collected by the software. Experiments were performed with eight mice per group, and data acquisition was blinded.

### Data Analysis.

All datasets were processed in GraphPad PRISM version 7.05 and are presented as mean ± SEM. Data were statistically analyzed using Mann–Whitney *U* test, unless otherwise stated: *P < 0.05, **P < 0.01, ***P < 0.001, and ****P < 0.0001.

## Supplementary Material

Supplementary File

## Data Availability

All study data are included in the article and/or *SI Appendix*.
