# Supplementary material for: Elimination of fibrin γ-chain cross-linking by FXIIIa increases pulmonary embolism arising from murine inferior vena cava thrombi
Source: Proc Natl Acad Sci U S A. 2021 Jun 28;118(27):e2103226118. doi: 10.1073/pnas.2103226118 (PMC8271579; doi:10.1073/pnas.2103226118)
Supplement: Supplementary File [file pnas.2103226118.sapp.pdf]

PNAS
www.pnas.org

Supplementary Information for

Elimination of fibrin γ-chain cross-linking by FXIIIa increases pulmonary embolism arising from murine inferior vena cava thrombi

Duval C., Baranauskas A., Feller T., Ali M., Cheah L.T., Yuldasheva N.Y., Baker S.R., McPherson H.R., Raslan Z., Bailey M.A., Cubbon R.M., Connell S.D., Ajjan R.A., Philippou H., Naseem K.M., Ridger V.C., Ariëns R.A.S.*

*** Corresponding Author:**
Professor Robert A. S. Ariëns
R.A.S.Ariens@leeds.ac.uk

This PDF file includes:
Supplementary text
Figures S1 to S6
Tables S1
SI References

SI METHODS

Whole Blood Clot Contraction

Clot contraction was analysed using a protocol adapted from Aleman *et al.* (1). 7.5 mm diameter flat bottom glass tubes (Bio/Data Corp) were siliconised with Sigmacote for 10 min at RT, rinsed with ddH_2O and left to dry. 460 μl of diluted whole blood (one-fourth in isotonic saline) was transferred to the siliconised tube, and clotting was initiated by adding 40 μl of activation mix (15 μl of PPP-Reagent [Thrombinoscope B.V.], 5 μl of 1 M $CaCl_2$, 20 μl of isotonic saline). Clots were left to form and contract at 37 °C over 2 h, with photographs taken at time-points 0, 30, 60, 90, and 120 min. After 120 min, clots were carefully removed from the tubes and weighed using a fine balance. Clot volumes over time were determined using ImageJ software v1.47 (National Institutes of Health), assuming a cylinder shape. Experiments were performed in triplicate, for 8 mice per group.

Washed Platelets Clot Retraction

Clot retraction was measured using a protocol adapted from Buitrago et al. (2). PRP was obtained by centrifugation of whole blood supplemented with 500 μl MTB at 100 *g* for 5 min at room temperature. The resulting pellet was resuspended in MTB, supplemented with 0.2 U/ml apyrase and 10 μM indomethacin, and re-centrifuged at 400 *g* for 10min, the washed platelet pellet was resuspended in MTB and cells were counted. Clot retraction was performed in siliconised tubes using 200μl of washed platelets (2 x 10^8 plt/ml), thrombin (0.3 U/ml) and $CaCl_2$ (10 mM). Clots were left to form and retract at room temperature over 2hr, with photographs taken at time-points 0, 30, 60, 90, and 120 min. Final clot volumes over time were determined using ImageJ software, assuming a cylinder shape. Experiments were performed in duplicate, for 4 mice per group.

Inferior Vena Cava Stasis

Full ligation of the inferior *vena cava* was performed as previously described (1). Mice (8-wk-old) were anaesthetised with 1.5% isoflurane in oxygen (2 l/min flow rate), and the abdomen was prepared for sterile laparotomy. The bowels were exteriorised, and the inferior vena cava side and back branches were ligated with 8-0 prolene sutures. The infrarenal part of the inferior *vena cava* was bluntly separated from the aorta, distally to the renal branches, and the vessel fully ligated with 8-0 prolene suture. The bowels were moved back into the abdominal cavity, with saline, and the muscle and skin layers were sutured sequentially. Animals were immediately injected with 100 μl of 0.1 mg/ml Vetergesic for pain relief and allowed to recover. After 24 h, the animals were anaesthetised, blood was withdrawn by cardiac puncture using 10% (v/v) 0.109 M sodium citrate, for platelet-poor plasma preparation, and the thrombi were separated from the vein will before being weighed and fixed in 2.5% glutaraldehyde or 4% paraformaldehyde.

Lateral Atomic Force Microscopy

Preparation of substrate and clots

The striated substrate was made of cured optical adhesive as described previously (3, 4). Briefly, a polydimethylsiloxane stamp was prepared with a mixture of Sylgard dimethylsiloxane and catalyst (Dow), and poured over a silicone master. The stamp was then cured at 70 °C for 1 h, removed from the silicone master, pressed in optical glue (Thorlabs), and cured for 3min under ultraviolet (UV) light (λ = 365 nm). The stamp/substrate was then carefully removed and stored in 2% sodium dodecyl sulfate. For the experiments, substrates with 6-μm-wide ridges, 10-μm-wide and 6-μm-deep channels were prepared.

Clotting mixtures (17.5 μl) containing fibrinogen (0.5 mg/ml), zymogen FXIII (0 or 10 μg/ml), thrombin (0.5 U/ml) and $CaCl_2$ (5 mM) were quickly placed on the striated surface and incubated for > 1 h in a humid chamber. The clots were then gently washed with TBS, incubated for 10 min with 20-nm-yellow-green carboxylate FluoSpheres (Thermo Fisher Scientific), rinsed, and stored in TBS.

Fibre manipulation

A MFP3D atomic force microscope (Oxford Instruments) combined with an Axiovert 200 optical/fluorescence microscope (Zeiss), CSC38 cantilevers (MikroMasch), and a custom-written extension of the Asylum software (cantilever movement in the *x*-, *y*- and *z*- direction, recording of *x*-, *y*-, *z*- displacement and lateral signal) were used to measure the mechanical response of individual fibrin fibres upon lateral stretching. Individual fibres were pulled by the cantilever at a rate of 100 nm/s, while the deformation was visualised and followed with the fluorescence microscope. Fibres were pulled until rupture, and those that slipped on the ridge were excluded from the analysis. Pulled fibres were scanned in contact mode on the ridge, and the width and height of the fibres were measured.

Stress and strain calculations

The lateral force was calculated from the lateral deflection of the cantilever based on the Liu method (5). The inverse optical lever sensitivity ($invOLS$ in m/V) and the spring constant (k_n) of the cantilever were measured before each set of experiments. The $invOLS$ was the slope of the contact region of deflection against displacement curves registered on a clean glass surface, while the spring constant of the cantilever was determined using the Thermal Noise Method from the Asylum software (v16, Oxford Instruments).

Lateral force (F_l) was calculated from the lateral deflection signal (Δx) using the effective spring constant of the cantilever,

$$F_l = k_c \cdot \Delta x$$

The effective spring constant (k_c) was calculated from the $invOLS$, width (w), length (l), thickness (t) of the cantilever, height of the tip (h) and Young's modulus of silicon (E = 1.69×10^{11} N/m²),

$$k_c = \frac{w \cdot t^3 \cdot E}{6 \cdot l^2 \cdot (h + t/2)} \cdot \frac{1}{invOLS}$$

where the cantilever thickness was calculated from the measured spring constant.

$$k_n = \frac{E * w * t^3}{4 * l^3}$$

Lateral force (F_l) was the force exerted on and detected by the cantilever. The cantilever was positioned next to the fibre in the middle of the channel (at 5 μm distance from both ridges), thus the force exerted on the fibre by the cantilever (F_{fibre}) could be calculated from F_l and the ratio between the pulling distance (s) and the half of the well width ($5\ \mu m$), which was equal to the original half-length of the strained fibre,

$$F_{fibre} = \frac{F_l}{2 * \sin\left(\tan^{-1}(s/5\ \mu m)\right)}$$

Dividing F_{fibre} with the cross-sectional area resulted in the engineering stress ($\sigma = F_{fiber}/A$) exerted on the fibre. The cross-sectional area of each fibre was calculated as the area of an ellipse from the height and the width obtained from scanning each fibre,

$$A = width/2 \cdot height/2 \cdot \pi$$

The extension of the fibres was calculated as normal strain: amount of deformation (δ) per unit original length ($5\ \mu m$). The amount of deformation was the actual length of the fibre (L') minus the original length ($5\ \mu m$),

$$\varepsilon = \frac{\delta}{5\ \mu m} = \frac{L' - 5\ \mu m}{5\ \mu m}$$

Where L' was calculated from the pulling distance (s) and the half of the well width ($5\ \mu m$),

$$L' = \sqrt{s^2 \cdot 5^2}$$

Tangent modulus calculations

To obtain information about the stiffness of the fibres at any strain value, each stress-strain curve (***SI Appendix*, Fig. S6 A**) was differentiated (1st order derivative) into tangent modulus vs strain (***SI Appendix*, Fig. S6 B**). As the strain increment between data points was relatively small (around 0.00002 at small strains and < 0.003 at large strains), the differentiated curve showed fluctuations. These spikes were smoothed with a median filter for a 50 data point window (***SI Appendix*, Fig. S6 B**).

The tangent modulus shows the momentary stiffness of the fibre at each strain. The tangent modulus was relatively constant at strains up to 1.5, increased between 1.5-2.5, reached a plateau at a maximum value before rupture and decreased at rupture. Tangent moduli were measured for each fibre strain values of 0.5, 1.0, 2.5 and 3.0, averaged and plotted (***SI Appendix*, Fig. S6 C**). The averaged tangent moduli were similar for strains 0.5 and 1 in each fibrinogen variant, and increased for both variants for strains 2.5 and 3. The start of the strain stiffening was slightly different for each fibre, and fibres ruptured at different strains (ε_{max} = 309±49). Nevertheless, each group showed a similar average for tangent modulus at 0.5 and 1.0 strain (***SI Appendix*, Fig. S6C**), and there was good agreement between slope1 values and the tangent modulus obtained at a strain of 1.0 (***SI Appendix*, Table S1**). However, as fibres

ruptured at different strains, tangent modulus values at strains of 2.5 and 3.0 differed from slope2, which was measured immediately before the rupture for each fibre (***SI Appendix*, Table S1**).

Thus, for the analysis of the stiffness, the tangent modulus was not used, but the slopes for the linear part of each curve at low strain (<1.5, slope1) and before rupture (slope2) were calculated and used for further analysis.

Analysis

For each fibre, stress (σ) vs strain (ε) curves were plotted and analysed. The following parameters were estimated from the curves:

- *Slope 1* (*s1*) [MPa]: Local slope of the first linear part of the curve under 1.5 strain. Value of fibre stiffness at low strains, assuming a linear relationship. As the onset of the strain stiffening slightly varied for each fibre, *s1* was measured instead of using the tangent modulus at a given strain.
- *Slope 2* (*s2*) [MPa]: Highest slope at the linear part of the curve before rupture. Value of fibre stiffness at high strains, assuming a linear relationship. As fibres ruptured at different strains, *s2* was measured instead of using the tangent modulus at a given strain.
- *h* (*s2*/*s1*): Ratio of slope 2 to slope 1. Gives the amount of strain stiffening: how much the initial stiffness increased until the fibre rupture.
- *Stress max* [MPa]: Maximal engineering stress, where the fibre ruptured.
- *Toughness* [MPa or MJ/m^3]: Area under the stress-strain curve, obtained by integration. Providing the amount of energy per unit volume required to produce failure, thus the resistance of the fibre to rupture.

```
hFGG   MSWSLHPRNLILYFYALLFLSSTCVAYVATRDNCCILDERFGSYCPTTCGIADFLSTYQT   34 (mature seq.)
mFGG   MSWSLQPPSFLLCCV-LLLFSPTGLAYVATRDNCCILDERFGSFCPTTCGIADFLSSYQT   34 (mature seq.)
       *****:* .::*    **::* * :******************:************:***

hFGG   KVDKDLQSLEDILHQVENKTSEVKQLIKAIQLTYNPDESSKPNMIDAATLKSRKMLEEIM   94
mFGG   DVDNDLRTLEDILFRAENRTTEAKELIKAIQVYYNPDQPPKPGMIDSATQKSKKMVEEIV   94
       .**:**::*****.:.**:*:*.*:******: ****:  **.***:** **:**:***:

hFGG   KYEASILTHDSSIRYLQEIYNSNNQKIVNLKEKVAQLEAQCQEPCKDTVQIHDITGKDCQ  154
mFGG   KYEALLLTHETSIRYLQEIYNSNNQKITNLKQKVAQLEAQCQEPCKDSVQIHDTTGKDCQ  154
       **** :***::****************.***:***************:***** ******

hFGG   DIANKGAKQSGLYFIKPLKANQQFLVYCEIDGSGNGWTVFQKRLDGSVDFKKNWIQYKEG  214
mFGG   EIANKGAKESGLYFIRPLKAKQQFLVYCEIDGSGNGWTVLQKRIDGSLDFKKNWIQYKEG  214
       :*******:******:****:******************:***:***:************

hFGG   FGHLSPTGTTEFWLGNEKIHLISTQSAIPYALRVELEDWNGRTSTADYAMFKVGPEADKY  274
mFGG   FGHLSPTGTTEFWLGNEKIHLISMQSTIPYALRIQLKDWNGRTSTADYAMFRVGPESDKY  274
       *********************** **:******::*:**************:****:***

hFGG   RLTYAYFAGGDAGDAFDGFDFGDDPSDKFFTSHNGMQFSTWDNDNDKFEGNCAEQDGSGW  334
mFGG   RLTYAYFIGGDAGDAFDGYDFGDDPSDKFFTSHNGMQFSTWDNDNDKFEGNCAEQDGSGW  334
       ******* **********:*****************************************

hFGG   WMNKCHAGHLNGVYYQGGTYSKASTPNGYDNGIIWATWKTRWYSMKKTTMKIIPFNRLTI  394
mFGG   WMNKCHAGHLNGVYHQGGTYSKSSTTNGFDDGIIWATWKSRWYSMKETTMKIIPFNRLSI  394
       **************:*******:** **:*:********:******:***********:*

hFGG   GEGQQHHLGGAKQAGDV  411
mFGG   GEGQQHHMGGSKQAGDV  411
       *******:**:******
```

Fig. S1. Protein sequence alignment of human and murine fibrinogen γ-chain. The 3 residues (highlighted in yellow) involved in γ-chain cross-linking in human fibrinogen are conserved in murine fibrinogen (Q398, Q399, K406 for both mature protein sequences). The underlined sequence indicates the signal peptide.

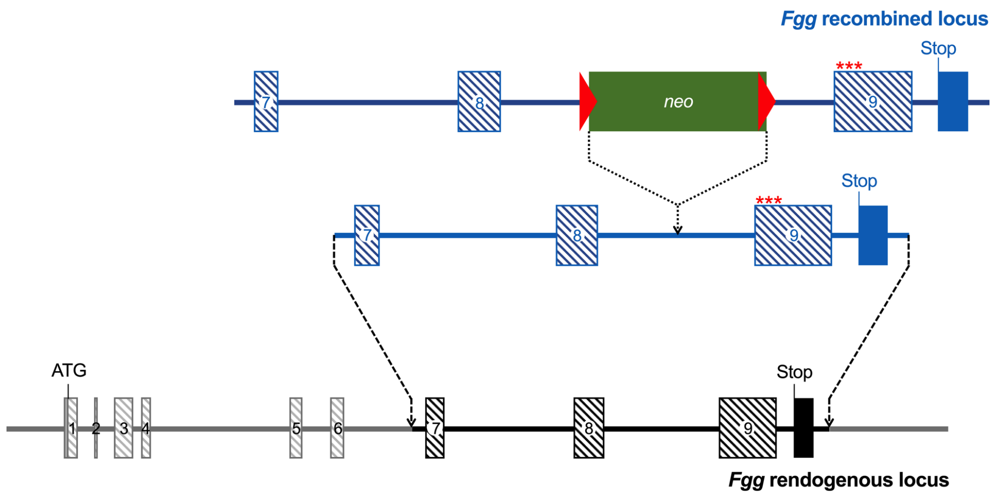

Fig. S2. Schematic representation of the selected targeting strategy for the generation of the FGG3X murine model. Hatched rectangles represent the exons of the Fgg coding sequences, plain rectangles indicate non-coding exon portions, solid lines represent chromosome sequences, whilst red stars represent the 3 mutated codons in exon 9. The neomycin positive cassette is indicated, surrounded by loxP sites represented in red. The initiation (ATG) and stop (Stop) codons are also indicated.

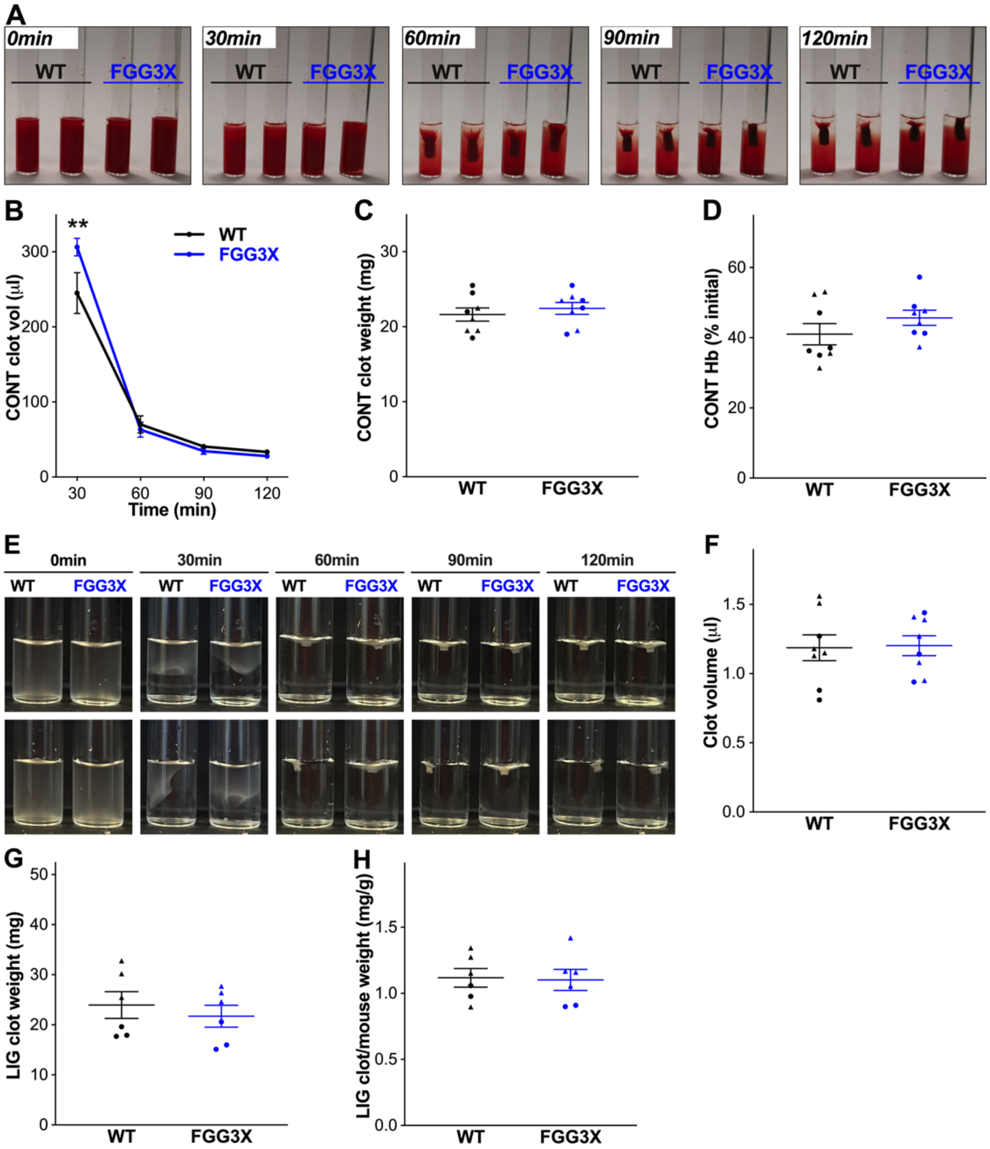

Fig. S3. Whole blood clot contraction, washed platelet clot retraction, and in-vivo thrombus weight are unaffected in FGG3X compared with WT mice. Whole blood clot contraction induced by PPP-reagent and $CaCl_2$ (3% and 10 mM) (*A*) showed that the reduction in clot volume over time (*B*) and final clot weight (*C*) were similar for FGG3X and WT mice. The amount of supernatant haemoglobin (*D*) was reduced over time to a similar extent in FGG3X and WT mice. Washed platelets ($2x10^8$/ml) clot retraction induced by thrombin and $CaCl_2$ (0.3 U/ml and 1 mM) (*E*) showed no difference in the retraction kinetic, resulting in a similar final clot volume for FGG3X and WT mice (*F*). Stasis experiments showed that 24 h after ligation of the inferior *vena cava*, raw weight (*G*) and normalised weight (*H*) of the thrombi formed in FGG3X and WT mice were similar. *n* = 8 (*A-D*), 4 (*E, F*) and 6 (*G-H*); [▲] males, [●] females. Data are presented as mean ± SEM, and analysed by Two-Way ANOVA test (*B*) and Mann-Whitney *U* test (*C, D, F-H*); ***P* < 0.01.

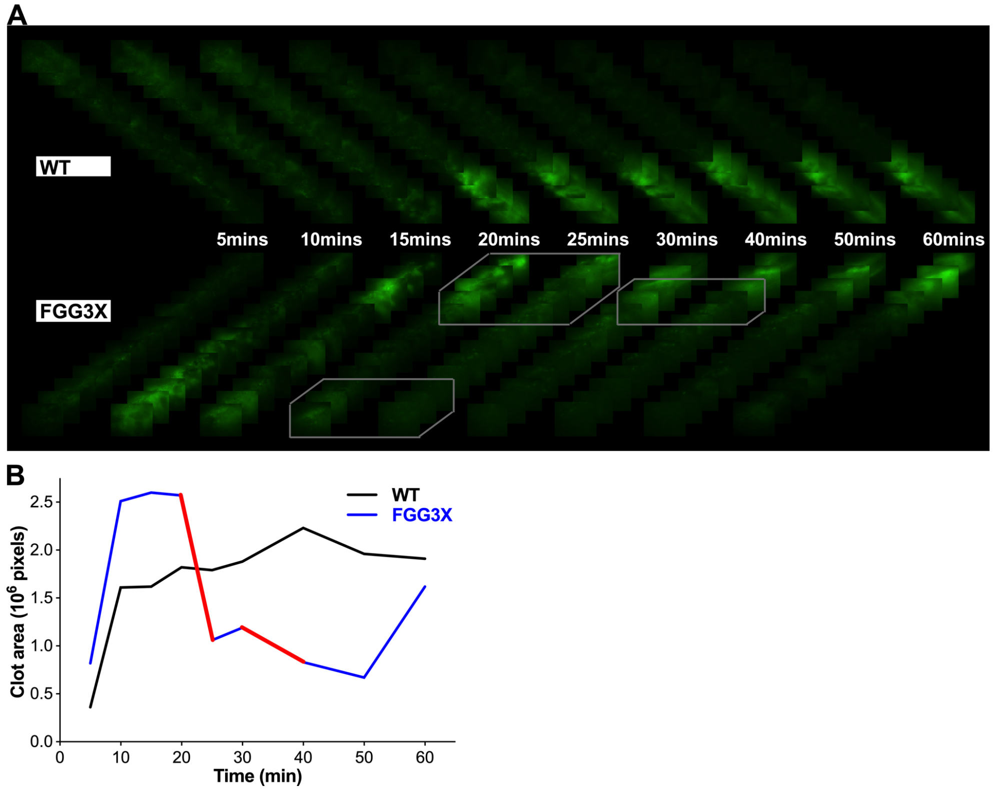

Fig. S4. Representative images from intravital analysis of venous clot formation. Following injection of 100 μg AlexaFluor[488]-fibrinogen and injury to the femoral vein with 10% $FeCl_3$ for 3 min, clot size was measured over time by intravital fluorescence microscopy (*A, B*) in WT and FGG3X mice. The grey boxes (*A*) show areas of marked decreased fibrinogen content and red lines (*B*) indicate a decrease in clot size by more than 25% over 5 min.

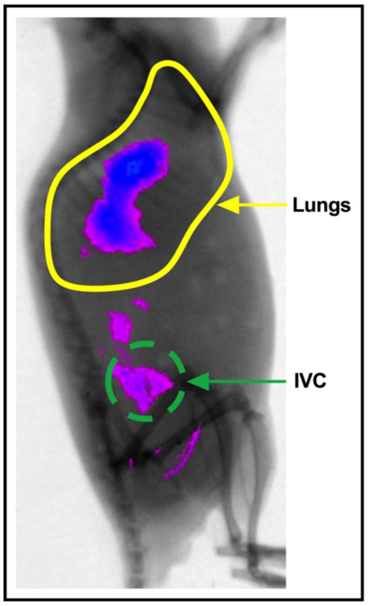

Fig. S5. Xtreme imaging quantification of pulmonary embolism. Using the X-ray layer, the rib cage containing the lungs (yellow) was delineated, and total fluorescence intensity was quantified using the ‘ROI free form’ tool (Molecular Imaging software; Bruker). The $FeCl_3$-induced thrombus in the inferior *vena cava*, from which the pulmonary emboli originate, is highlighted in green.

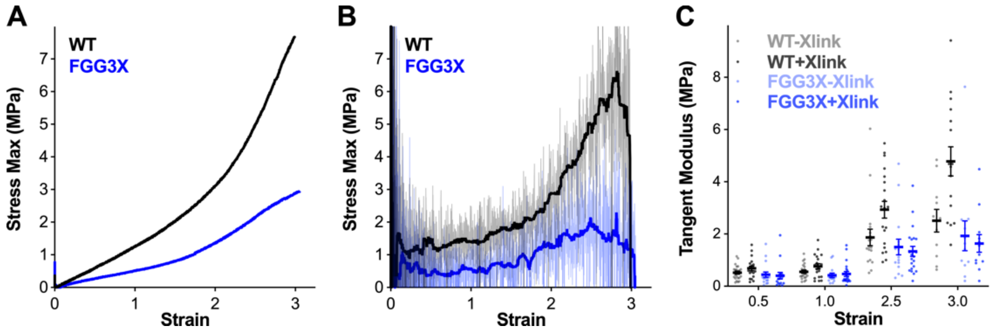

Fig. S6. Calculation of tangent modulus. Stress-strain curves of crosslinked FGG3X and WT fibre (*A*, representative) were differentiated into tangent modulus vs strain curves (*B*; dark line, median filter curves; light lines, row differentiated curves). The resulting tangent modulus data shows that WT crosslinked fibres were the stiffest at each strain. Averages of the tangent moduli for strain of 0.5 and 1 were similar for each condition, increased at strain of 2.5 but differed between strains 2.5 and 3 as some of the fibres ruptured within this range (*C*). *n* = 18-22. Data are presented as mean ± SEM.

Table S1. Comparison of tangent moduli and stress vs strain slopes

PARAMETERS	WT -Xlink	WT +Xlink	FGG3X -Xlink	FGG3X +Xlink
Tangent modulus *(strain = 1)*	0.56 ± 0.29	0.76 ±0.43	0.44 ± 0.40	0.47 ± 0.40
Slope 1	0.53 ± 0.28	0.74 ± 0.40	0.47 ± 0.39	0.42 ± 0.36
Tangent modulus *(strain = 2.5)*	1.87 ±1.30	2.93 ±1.41	1.50 ±1.17	1.33 ±0.86
Tangent modulus *(strain = 3)*	2.51 ± 1.37	4.79 ± 2.16	1.93 ± 1.88	1.64 ± 1.13
Slope 2	1.75 ± 1.45	4.38 ± 2.50	1.92 ± 1.66	1.40 ± 1.00

Values are in [MPa]. *n* = 18-22.

SI REFERENCES

1. M. M. Aleman *et al.*, Factor XIII activity mediates red blood cell retention in venous thrombi. *J. Clin. Invest.* **124**, 3590-3600 (2014).
2. C. L. Buitrago *et al.*, Dominant role of αIIbβ3 in platelet interactions with cross-linked fibrin fragment D-dimer. *Blood Adv.* **4**, 2939-2949 (2020).
3. S. Baker *et al.*, The mechanical properties of dry, electrospun fibrinogen fbers. *Mater. Sci. Eng. C Mater. Biol. Appl.* **32**, 215-221 (2012).
4. C. C. Helms, R. A. Ariens, S. Uitte de Willige, K. F. Standeven, M. Guthold, alpha-alpha cross-links increase fibrin fiber elasticity and stiffness. *Biophys. J.* **102**, 168-175 (2012).
5. W. Liu, K. Bonin, M. Guthold, Easy and direct method for calibrating atomic force microscopy lateral force measurements. *Rev. Sci. Instrum.* **78**, 063707 (2007).
